# Repetition Suppression and Memory for Faces is Reduced in Adults with Autism Spectrum Conditions

**DOI:** 10.1093/cercor/bhw373

**Published:** 2016-11-30

**Authors:** Michael P. Ewbank, Philip J. Pell, Thomas E. Powell, Elisabeth A. H. von dem Hagen, Simon Baron-Cohen, Andrew J. Calder

**Affiliations:** 1 Medical Research Council, Cognition and Brain Sciences Unit, CB2 7EF Cambridge, UK; 2 School of Psychology, Cardiff University, CB2 8AH Cardiff, UK; 3 Department of Psychiatry, Autism Research Centre, University of Cambridge, CF10 3AT Cambridge, UK

**Keywords:** autism, face-memory, fMRI-adaptation, fusiform, objects

## Abstract

Autism spectrum conditions (ASC) are associated with a number of atypicalities in face processing, including difficulties in face memory. However, the neural mechanisms underlying this difficulty are unclear. In neurotypical individuals, repeated presentation of the same face is associated with a reduction in activity, known as repetition suppression (RS), in the fusiform face area (FFA). However, to date, no studies have investigated RS to faces in individuals with ASC, or the relationship between RS and face memory. Here, we measured RS to faces and geometric shapes in individuals with a clinical diagnosis of an ASC and in age and IQ matched controls. Relative to controls, the ASC group showed reduced RS to faces in bilateral FFA and reduced performance on a standardized test of face memory. By contrast, RS to shapes in object-selective regions and object memory did not differ between groups. Individual variation in face-memory performance was positively correlated with RS in regions of left parietal and prefrontal cortex. These findings suggest difficulties in face memory in ASC may be a consequence of differences in the way faces are stored and/or maintained across a network of regions involved in both visual perception and short-term/working memory.

## Introduction

Recognizing faces is vital for social functioning, enabling us to engage in appropriate interactions and to form social bonds. Individuals with autism spectrum conditions (ASC)—a heritable condition associated with difficulties in social communication, unusually narrow interests and repetitive behaviors (American Psychiatric Association 2013)—show difficulties in processing facial identity, gaze, and expression (Harms et al. 2010; Weigelt et al. 2012). Such impairments have been proposed to lead to social difficulties experienced by those with the condition (Schultz 2005). A recent review (Weigelt et al. 2012) indicates that difficulties in face-identity recognition in ASC are primarily found on tasks that involve a memory component (i.e., a delay between target and test stimulus), whereas measures of face identity “perception” (e.g., the face-inversion effect) appear similar to neurotypical controls.

Previous work investigating the neural mechanisms of face processing in ASC has primarily focused on the magnitude of the response in a region of occipitotemporal cortex known as the fusiform face area (FFA). While some studies report a reduced FFA response in ASC participants (Schultz et al. 2000; Pierce et al. 2001; Hall et al. 2003) other work either found no group differences (Hadjikhani et al. 2004; Pierce et al. 2004) or that differences are eliminated when controlling for fixation on the eye region of the face (Dalton et al. 2005). To date, no studies have investigated repetition suppression (RS) to faces in FFA in individuals with ASC. RS refers to the decrease in neural activity observed when a stimulus is presented repeatedly, relative to when different stimuli are presented (Grill-Spector et al. 2006). Numerous studies have shown that viewing repeated presentations of the same face leads to RS in the FFA in neurotypical populations (Grill-Spector and Malach 2001; Andrews and Ewbank 2004). At a behavioral level, evidence indicates that face aftereffects—the bias in perception following prolonged exposure to a stimulus—are significantly reduced in children with autism and in first-degree relatives (Pellicano et al. 2007; Fiorentini et al. 2012; Pellicano et al. 2013), although such differences have not been found in adults (Cook et al. 2014; Walsh et al. 2015). However, while aftereffect paradigms involve prolonged exposure to a stimulus, RS paradigms typically involve brief presentations of faces separated by a delay, and are therefore more similar to behavioral tests of face memory. In Experiment 1, we measured RS to faces in face-selective regions of occipitotemporal cortex in a group of adults with a clinical diagnosis of an ASC and in a group of age and IQ matched controls. Given our previous finding, that RS to faces in FFA is reduced as a function of increasing numbers of autistic traits in a neurotypical sample (Ewbank et al. 2014), here we predicted that individuals with ASC would showed reduced RS to faces in FFA relative to controls.

The second aim of our study was to determine whether any reduction in RS in ASC participants is specific to faces, or generalizes to other (non-social) stimulus categories. Previously, we found that the relationship between diminished RS and increasing autistic traits extended to non-face categories (scenes and geometric shapes) (Ewbank et al. 2014), suggesting the relationship between RS and autistic traits could reflect differences in the mechanisms underlying RS in low and high autistic trait participants. The precise mechanisms underlying RS are unclear; however, models of predictive coding propose that RS reflects the match between top-down (prediction-based) and bottom-up (stimulus-based) inputs, with predictions serving to suppress responses (or errors) to incoming sensory information (Rao and Ballard 1999; Henson 2003; Friston 2005). Thus, consecutive occurrence of the same face should lead to a reduction in prediction error, and a subsequent reduction in neural activity. Given that atypicalities in autism have recently been characterized as a “disorder of prediction” (Sinha et al. 2014) or “attenuated use of prior knowledge” (Pellicano and Burr 2012) (see also Mitchell and Ropar 2004), the proposed role of prediction in RS may be of particular relevance to understanding reduced RS in ASC. If diminished RS in ASC reflects atypicalities in “predictive” mechanisms, then we would expect reduced RS to both faces and non-faces in ASC participants. To address this, in Experiment 2 we measured RS to simple geometric shapes in object-selective regions of occipitotemporal cortex.

Finally, we also investigated the relationship between RS and memory for faces. Previous studies report that adults with ASC show reduced performance on a standardized test of face memory relative to controls (O'Hearn et al. 2010; Kirchner et al. 2011); however, the extent to which such memory difficulties are specific to faces is unclear, given that this work did not include equivalent tests of non-face memory. Similarly, while imaging work indicates individuals with ASC show reduced activity in parietal and prefrontal regions when performing a face working memory task (Koshino et al. 2008), the absence of an equivalent non-face task makes it difficult to infer whether this difference reflects a specific impairment in face memory or a more general deficit in visual working memory. In order to address the face-selective nature of memory difficulties in ASC, and their relationship with RS, we obtained measures of participants’ performance on standardized tests of face memory (Duchaine and Nakayama 2006) and car memory (Dennett et al. 2012), matched in format and memory demands.

## Materials and Methods

### Participants

16 neurotypical volunteers and 17 individuals with a clinical diagnosis of autism or Asperger syndrome participated in the study. One control participant was excluded after scoring high on the Autism Spectrum Quotient (AQ) and Social Responsiveness Scale (SRS) (borderline clinical range). One ASC participant was excluded after scoring within the control range on both measures. The data from another ASC participant were removed due to excessive head movement in the scanner (>4 mm). This left a total of 30 participants (15 per groups). Groups were matched for age, sex, and IQ (see Table 1). Neurotypical participants were recruited through the MRC Cognition and Brain Sciences Unit's research participation system. Participants with ASC were recruited via the Cambridge Autism Research Database, and had written confirmation of an independent diagnosis of an ASC (autism or Asperger syndrome) by a qualified clinician using DSM-IV criteria (American Psychiatric Association 2000). In addition, 6 previously had their diagnosis confirmed via the Adult Asperger Syndrome Assessment (Baron-Cohen et al. 2005), 4 using the Autism Diagnostic Observation Schedule (Lord et al. 2000), and 2 via the Autism Diagnostic Interview-Revised (Lord et al. 1994).

**Table 1:** Demographics for all control and ASC participants included in the study

	Controls	ASC	Comparison
*N*	15	15	
Sex (*n* male: *n* female)	9:6	10:5	*P *= 0.62
Age (years)			
Mean (SD)	28.1 (7.5)	31.8 (9.2)	*P *= 0.23
Range	19–40	18–45	
AQ			
Mean (SD)	11.7 (4.1)	40.5 (6.8)	*P *< 0.001
Range	4–17	30–49	
SRS			
Mean (SD)	25.3 (15.4)	108 (25.8)	*P *< 0.001
Range	10–50	60–133	
Full-scale IQ			
Mean (SD)	128.2 (10.5)	126 (11.8)	*P *= 0.76
Range	113–141	99–143	

All participants completed the Wechsler Abbreviated Scale of Intelligence—second edition (Wechsler 2011) and all scored >99. All participants were right-handed, had normal or corrected to normal vision and were naive to the aims of the experiment. No participants were taking psychotropic medication at the time of the study or had a current psychiatric diagnosis (except ASC). Participants completed the AQ (Baron-Cohen et al. 2001) and the SRS (Constantino and Gruber 2005), a quantitative measure for identifying ASC symptoms in neurotypical and clinical adult populations. The study was approved by the Cambridge Psychology Research Ethics Committee. All volunteers provided written informed consent and were paid for participating.

### Behavioral Tests

Before scanning, all participants completed the Cambridge Face Memory Test (CFMT) (Duchaine and Nakayama 2006) and the Cambridge Car Memory Test (CCMT) (Dennett et al. 2012). The CFMT and the CCMT assess recognition of unfamiliar faces and cars, respectively. Both tests use identical formats, comprising 72 trials. During a training phase, participants are required to learn exemplars before being required to identify the trained exemplars in a 3 alternative forced choice procedure. As the test progresses, difficulty is increased by presenting items from different viewpoints (compared with the learned exemplar) and through the addition of visual noise.

Following the scanning session, participants performed an identity discrimination task, during which they were required to respond, via a button press, as to whether 2 consecutively presented faces were of the same or a different identity. Face images were identical to those used in the scanning session. Each trial consisted of consecutive images of 2 same or different identity faces, shown at the same or different sizes. Each image was shown for 1050 ms followed by a 200 ms blank ISI (identical to the procedures used in the scanning session). The experiment comprised 64 trials (32 same-identity, 32 different-identity).

### Stimuli

For the localizer scan and RS Experiment 1, color photographs of unfamiliar faces with neutral expressions were obtained from the NimStim (Tottenham et al. 2009), Karolinska Directed Emotional Faces (Lundqvist and Litton 1998) and FERET (Phillips et al. 2000) image sets. All face images were matched for interocular distance and eye position. Images of 8 simple geometric shapes were generated using Microsoft PowerPoint 2010 and Adobe Photoshop (http://www.adobe.com).

### Localizer Scan

Participants lay supine in the magnet bore and viewed images projected onto a screen visible via an angled mirror. The localizer scan comprised images of 64 household objects, 64 scrambled versions of the objects, 64 unfamiliar faces, and 64 scenes. These were presented using a block design, consisting of four 16 s blocks for each of the 4 conditions; each block contained 8 images with each image shown for 1600 ms followed by a 400 ms blank ISI. Blocks of stimuli were separated by an 8 s rest block (fixation). To ensure participants were attending to all trials in the localizer scan they performed a one-back matching task and responded, via a button press, whenever they saw the same image appear on 2 consecutive trials. All participants completed experimental runs in the following order: Experiment 1, Localizer, Experiment 2.

For each participant, face-selective FFA, occipital face area (OFA), and superior temporal sulcus (STS) were identified using the contrasts faces > scenes. Object-selective lateral occipital (LO) and posterior fusiform gyrus (pFs) were identified using the contrast objects > scrambled-objects. ROIs were identified using a minimal threshold of *P* < 0.01 uncorrected (10 contiguous voxels).

### RS experiments

**Figure 1.**
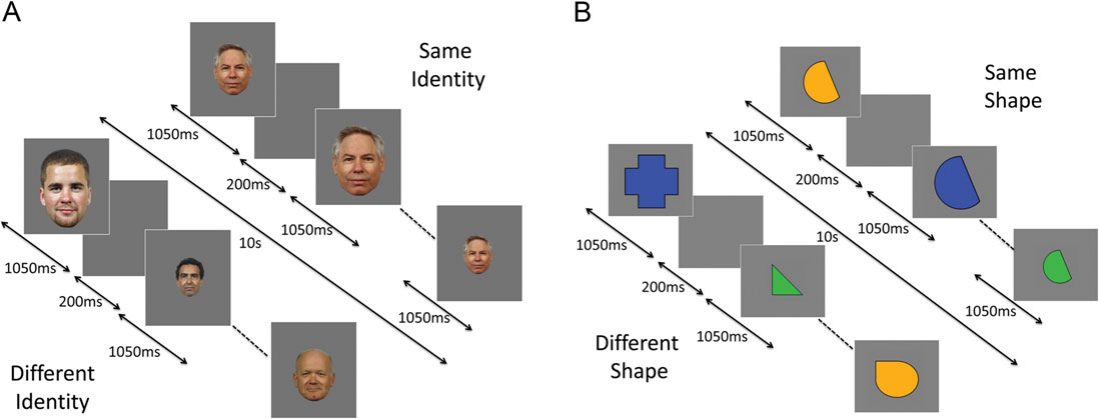
Block-design format used in (*A*) Experiment 1 (RS to faces) and (*B*) Experiment 2 (RS to shapes).

Experiment 1 used a repeated measures design, investigating the effect of Repetition (same-identity, different-identity) and Image Size (same-size, vary-size) (Fig. 1*A*). Same- and vary-size conditions each contained 8 same-identity blocks in which the same face was shown 8 times, and 8 vary-identity blocks containing images of 8 different faces; a total of 32 stimulus blocks. Each stimulus block lasted for 10 s, with each image shown for 1050 ms followed by a 200 ms blank ISI (Fig. 1*A*). In the same-size blocks, all faces subtended a visual angle of approximately 5.5° × 3.5°. Blocks in the vary-size condition contained images shown at regular-size and at 33% larger and 33% smaller than this size. Blocks were separated by an 8 s period of fixation when an equiluminant gray screen was shown. A total of 8 faces were used across the Experiment, and individual identities were shown an equal number of times in same- and vary-identity blocks. Total scan time was 9.6 min.

Experiment 2 used a repeated measures design investigating the effect of Repetition (same-shape, different-shape) and Image Color (same-color, vary-color) (Fig. 1*B*). Parameters were identical to those used in Experiment 1, with the exception that image size varied in all blocks (as in Ewbank et al. 2014). To test the possibility that reduced RS is due to ASC participants attending to different aspects of the stimulus on successive presentations, we included blocks in which repeated (and different) shapes were shown in the same color or in different colors. Individual shapes were shown an equal number of times in the same and different shape blocks. In both experiments, blocks were presented in a pseudorandomized order counterbalanced across participants. Participants performed a target detection task and responded, via a button press, whenever they saw a dot appear on an image (~15% of trials) in both experiments. To control for effects of target trials, the number of target trials and the location of dots on target trials were matched across the same- and different-identity blocks.

### Eyetracking

To determine whether participants were attending to the faces, we monitored and recorded participants’ eye movements during the scanning session using a 50 Hz monocular magnetic resonance imaging (MRI)-compatible infrared eyetracker (SensoMotoric Instruments, SMI). Eyetracking data were analysed with SMI BeGaze3.0 software. A rectangular area-of-interest (AOI) was created around the inner part of the upper face for each 3 image sizes separately. Average dwell time in the AOI was measured for each condition (excluding target trials). During scanning, participants were instructed to attend to a fixation cross which appeared during each ISI, and was positioned in a location equivalent to the center of the eyes.

### Imaging Parameters

MRI scanning was performed on a Siemens Tim Trio 3-Tesla MR scanner with a 32-channel head coil. Brain data were acquired with T2*-weighted echo-planar imaging sensitive to BOLD signal contrast. Each image volume consisted of 32 3 mm thick slices (voxel size 3 × 3 × 3 mm; slice gap 25%; FOV 192 × 192 mm; flip angle 78°; time echo 30 ms; time repetition 2 s). Slices were acquired sequentially in an oblique axial orientation aligned along the ventral temporal lobes. The first 3 volumes were discarded to allow for the effects of magnetic saturation. A high-resolution structural magnetization prepared rapid gradient echo scan was also acquired at a resolution of 1 × 1 × 1 mm. Following a scanner upgrade, 2 participants (1 ASC) were scanned (with an identical acquisition sequence) using a Siemens MAGNETOM Prisma-fit 3-Tesla MR scanner with a 64-channel head coil. To model any effects of the upgrade, scanner was included as a covariate in all analyses.

### fMRI Analysis

Data were analysed using SPM 8 software (Wellcome Trust Centre for Neuroimaging). Standard pre-processing was applied, including correction for slice-timing and head motion. Each participant's scans were normalized using the linear and nonlinear normalization parameters estimated from warping the participant's structural image to the Montreal Neurological Institute (MNI)—ICBM avg152 T1 weighted template, using 2 mm isotropic voxels and smoothed with a Gaussian kernel of 8 mm full-width half-maximum. Blocks of each condition were modeled by sustained epochs of neural activity (boxcars) convolved with a canonical hemodynamic response function. Realignment parameters were included as effects of no interest to account for motion-related variance. A high pass filter of 128 s was used to remove low-frequency noise.

### ROI Analysis

Mean parameter estimates for each condition were extracted from an 8 mm radius sphere centred on the maximal voxel in each participant's FFA, OFA, and STS (Experiment 1) and LO and pFs (Experiment 2) using MarsBar (Brett et al. 2002). In Experiment 1, parameter estimates for each ROI were entered into ANCOVAs including Repetition (same-identity, different-identity) and Image Size (same-size, vary-size), as repeated measures factors with Group (Control, ASC) as a between-participants factor and scanner as a covariate. Experiment 2 included Repetition (same-shape, different-shape) and Image Color (same-color, different-color) as repeated measures factors. RS was defined as a greater response to different-identity/shape conditions relative to same-identity/shape conditions. To examine the influence of autistic traits on RS to faces and shapes, we performed additional ANCOVAs for each ROI including AQ scores as a covariate.

### Whole Brain Analysis

In both experiments we determined whether regions outside of the category-selective ROIs showed a group difference in RS by performing an exploratory whole brain analysis. First-level images of contrast estimates (Different-Identity/Shape > Same-Identity/Shape) were entered into an independent samples* t*-test to compare RS between groups (*P* < 0.001 uncorrected, 10 contiguous voxels). To determine the relationship between individual variation in face memory and RS to faces, first-level images of contrast estimates (Different-Identity > Same-Identity) were entered into whole-brain regression analysis with CFMT and CCMT scores as covariates (*P* < 0.001 uncorrected, 10 contiguous voxels). Scanner was included as a covariate of no interest in all whole brain analyses.

## Results

### Behavioral Tests

**Figure 2.**
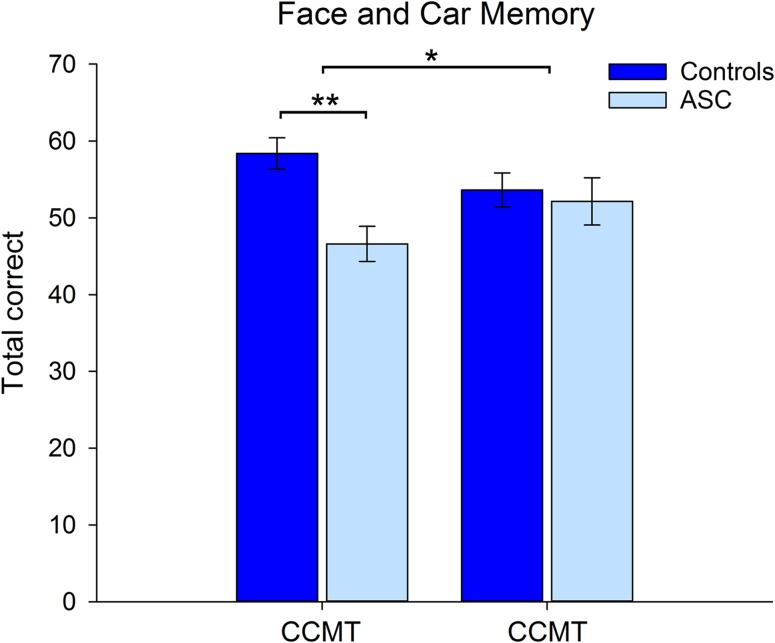
Accuracy data (±1 SE) for the Control and ASC group on the CFMT and the CCMT. *P < 0.05, **P < 0.001.

Face and car memory: Accuracy scores for the CFMT and CCMT were entered into a mixed-model ANOVA with Category (Face, Car) as a within-participants measure and Group (Control, ASC) as a between-participants factor. The results revealed a main effect of Group (*F*(1,28) = 5.77, *P* < 0.02, *η*_*ρ*_² = 0.17), reflecting greater overall accuracy scores in the control group. This effect was qualified by a significant interaction between Category and Group (*F*(1,28) = 5.42,* P* < 0.03, *η*_*ρ*_² = 0.16) (Fig. 2). Paired comparisons revealed that control participants showed greater performance on the face memory test relative to the ASC group (*t* (29) = 3.74, *P* < 0.005), with no group differences found on the car memory test (*P* = 0.80).

Face identity discrimination: Due to high accuracy rates in both groups (>96%), data were arcsine transformed before being entered into a 2 × 2 × 2 ANOVA (Identity × Size × Group). This revealed no significant effect of Group (*P* = 0.83) on accuracy, and no interactions between Identity and/or Image-Size and Group (*P*’s > 79). Accuracy (SD): Control = 96% (0.01); ASC = 96% (0.05).

### Imaging Experiments

Localizer scan: Mean MNI coordinates and numbers of face- and object-selective ROIs identified in both ASC and control groups are detailed in Table 2. The response to faces did not differ between groups in any face-selective ROI (*P*’s > 0.78).

**Table 2.** Mean MNI (±1 SD) coordinates and number of face- and object-selective ROIs identified in Control and ASC participants using the localizer scan

		Controls		ASC	
ROI	Hemi	*N*	MNI coordinates (SD)	*N*	MNI coordinates (SD)
Faces > Scenes					
FFA	R	15	41 (2.7), −46 (6.4), −20 (3.5)	13	41 (4.9), −43 (8.6), −18 (3.3)
					
	L	11	−39 (3.1), −48 (5.1), −21 (3.3)	11	−39 (3.8), −50 (5.4), −19 (4.1)
					
OFA	R	13	42 (5.0), −78 (8.1), −12 (5.0)	11	42 (5.8), −77 (7.0), −9 (5.7)
					
	L	10	−41 (3.7), −76 (7.6), −13 (6.3)	10	−43 (4.1), −77 (8.0), −13 (6.5)
					
STS	R	9	53 (7.9), −55 (5.8), 8 (5.0)	10	54 (7.5), −54 (6.6), 11 (3.4)
Objects > Scrambled					
LO	R	13	42 (4.3), −82 (6.6), −6 (4.7)	14	44 (5.8), −79 (6.2), −7 (6.5)
					
	L	13	−41 (4.0), −79 (6.8), −7 (5.8)	15	−42 (4.7), −79 (6.0), −7 (3.5)
pFs	R	12	36 (4.1), −43 (5.4), −20 (3.0)	10	36 (5.7), −43 (5.2), −19 (3.2)
	L	14	−37 (4.0), −48 (6.4), −18 (3.0)	13	−36 (4.8), −45 (7.5), −19 (1.9)

### Experiment 1: RS to Faces

**Figure 3.**
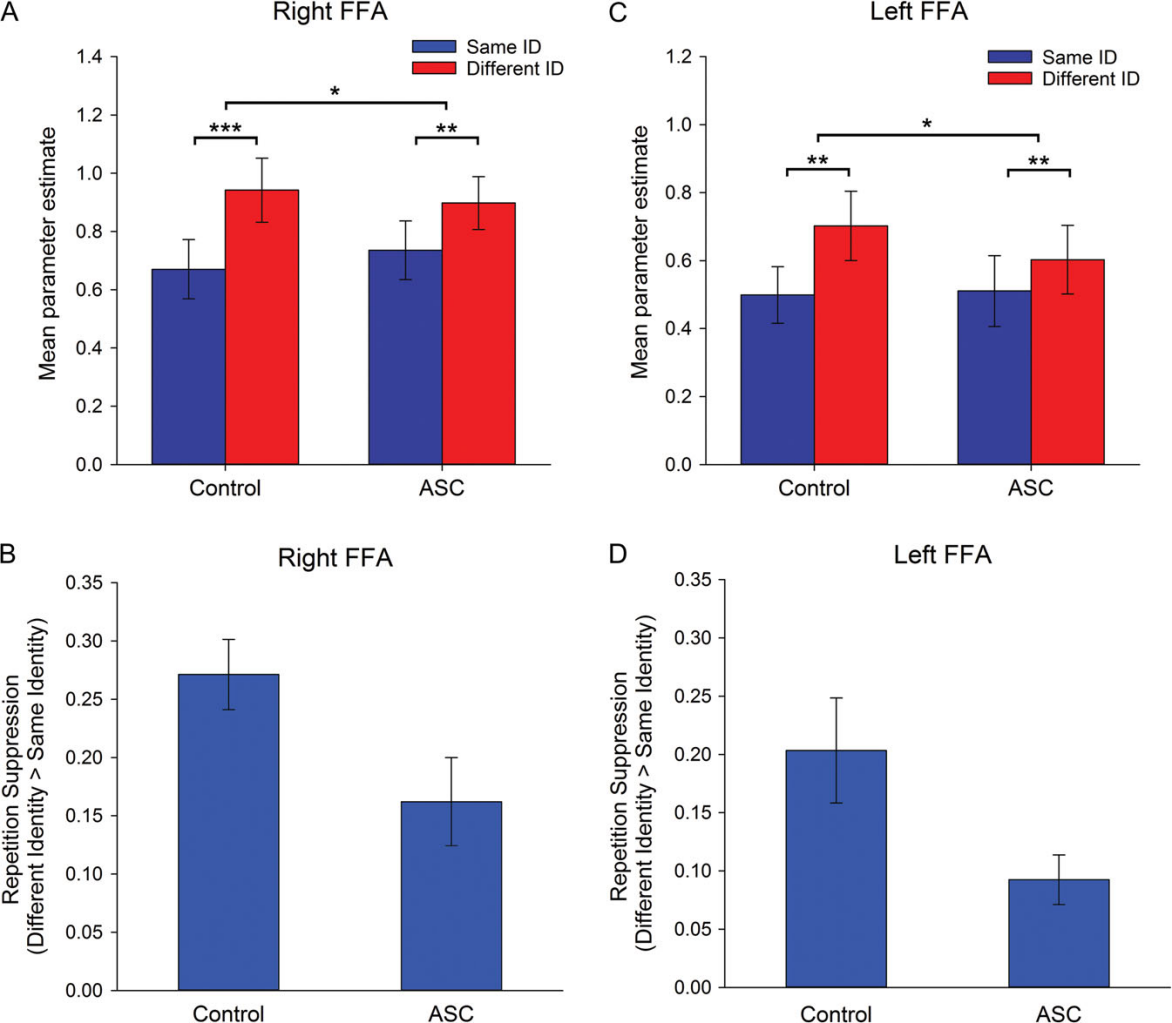
Experiment 1: RS to faces. Mean parameter estimates (±1 SE) for same- and different-identity conditions (across image-size) in (*A*) right FFA and (*C*) left FFA, in control and ASC participants. RS (±1 SE) (i.e., different identity–same identity) in (*B*) right FFA and (*D*) left FFA, in control and ASC participants. *P < 0.05, **P < 0.001, ***P < 0.0001.

Face-selective ROIs: For the right FFA, the ANCOVA revealed a significant effect of Repetition, reflecting a greater response in the different-identity condition compared with the same-identity condition (i.e., RS) (*F*(1,25) = 89.22,* P* < 0.001, *η*_*ρ*_² = 0.78), with no interaction between Repetition and Image-Size (*P* = 0.38). Crucially, there was a significant interaction between Repetition and Group in this region (*F*(1,25) = 5.94, *P* < 0.05, *η*_*ρ*_² = 0.19) (Fig. 3*A*). Paired comparisons revealed that the control group showed significantly greater RS than the ASC group *t*(26) = 2.44, *P* < 0.05) (Fig. 3*B*). This effect was not further modulated by an interaction with Image-Size (*P* > 0.87). There was no main effect of Group (*P* = 0.90), indicating the overall response to faces did not differ between groups.

In left FFA, an analogous ANCOVA revealed a significant effect of Repetition (*F*(1,19) = 30.08, *P* < 0.001, *η*_*ρ*_² = 0.61); with no interaction between Repetition and Image-Size (*P* = 0.79). Again, there was a significant interaction between Repetition and Group (*F*(1,19) = 4.76,* P* < 0.05, *η*_*ρ*_² = 0.19) (Fig. 3*C*), with controls showing greater RS than the ASC group (*t*(20) = 2.14, *P* < 0.05) (Fig. 3*D*). This effect was not further modulated by an interaction with Image-Size (*P *= 0.97), and again there was no main effect of Group (*P* = 0.75).

ANCOVAs for right and left OFA, revealed a main effect of Repetition in both regions: rOFA: (*F*(1,21) = 37.95, *P* < 0.001, *η*_*ρ*_² = 0.64); lOFA: (*F*(1,17) = 20.96, *P *< 0.001, *η*_*ρ*_² = 0.55), reflecting a greater response in the different-identity condition compared with the same-identity condition (see Supplementary Fig. 1), and no interaction between Repetition and Image Size (*P*’s > 0.37). There was no significant interaction between Repetition and Group in either right or left OFA: rOFA (*P* = 0.91); lOFA (*P* = 0.21), and no main effect of Group is either region (*P*’s > 0.66). Finally, an ANCOVA revealed no effect of Repetition in right STS (*P* = 0.42) and no interaction between Repetition and Group (*P* = 0.78). There was a significant effect of Group in this region (*F*(1,16) = 3.32, *P* = 0.05, *η*_*ρ*_² = 0.21), reflecting a greater response to faces in ASC group compared with the control group (see Supplementary Fig. 1).

Analogous ANCOVAs including AQ as a covariate across groups revealed a significant interaction between Repetition and AQ in right FFA (*F*(1,24) = 5.84, *P *< 0.05, *η*_*ρ*_² = 0.20) and left FFA *F*(1,18) = 4.10, *P* = 0.05, *η*_*ρ*_² = 0.19). This reflected diminishing RS as a function of increasing AQ scores. No significant interaction was found in other face-selective ROIs (*P*’s > 0.35).

#### Object-Selective ROIs

To determine whether any group difference in RS to faces was specific to face-selective ROIs, we examined RS to faces in object-selective regions (see Supplementary Fig. 2). An ANCOVA revealed a significant effect of Repetition in both right LO (*F*(1,24) = 37.91,* P* < 0.001, *η*_*ρ*_² = 0.61), and left LO (*F*(1,25) = 15.05,* P* < 0.001, *η*_*ρ*_² = 0.38), However, there was no interaction between Repetition and Group in either region (*P*’s > 0.76). ANCOVAs for right and left pFS, revealed a main effect of Repetition in both regions: right pFS: (*F* (1,19) = 24.81,* P *< 0.001, *η*_*ρ*_² = 0.57); left pFS: (*F*(1,25) = 35.22, *P* < 0.001, *η*_*ρ*_² = 0.59) and a significant interaction between Repetition and Group in both regions: right pFs: (*F*(1,19) = 4.26, *P* = 0.05, *η*_*ρ*_² = 0.18); left pFS: (*F* (1,25) = 7.04, *P *< 0.05, *η*_*ρ*_² = 0.23); with controls showing greater RS than the ASC group in both regions. Given that pFS shows considerable overlap with FFA and was defined using the contrast of objects > scrambled, it is likely that this region contains a large number of face-selective voxels. Thus, we performed an additional analysis using the contrast of objects > faces to define object-selective ROIs. Using this definition we again found significant RS to faces in all ROIs (*P*’s < 0.05), except left LO (*P* = 0.23), however, crucially, there was no interaction between Repetition and Group in either LO (*P*’s > 0.59) or pFs (*P*’s > 0.24). Thus, there were no group differences in RS to faces in object-selective ROIs after excluding face-selective voxels.

#### Whole Brain Analysis

There was no evidence of any group differences in RS at a whole brain corrected level (*P* < 0.05 FWE). At a more liberal threshold, and consistent with the ROI analysis, there was a group difference in a region corresponding to right FFA, with controls showing greater RS than the ASC group (*x* = 38, *y* = −54, *z *= −6,* t* = 3.51, *P* < 0.005 uncorrected).

### Relationship Between RS and Face Memory

To investigate the relationship between individual variation in face memory and RS in face-selective ROIs, data extracted from each ROI were entered into separate ANCOVAs examining the effects of Repetition and Image-Size as repeated measures, with CFMT and CCMT scores as covariates. This revealed no evidence of an interaction between RS and face memory in FFA or any other face-selective region (*P*’s > 0.11), although left OFA showed a borderline significant interaction between Repetition and CFMT (*F*(1,17) = 4.39, *P* = 0.05, *η*_*ρ*_² = 0.20), reflecting greater RS as a function of increased face memory scores.

**Figure 4.**
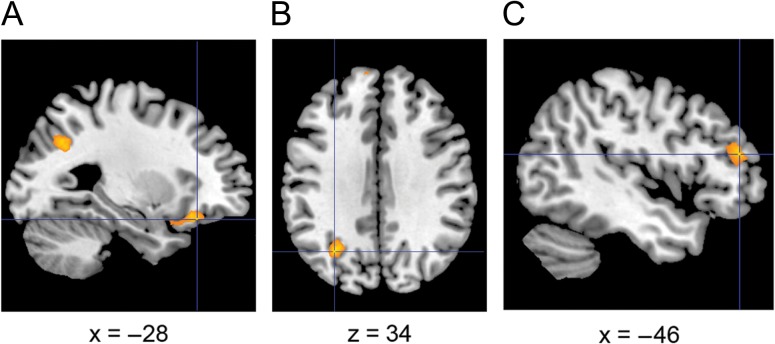
Activation maps showing positive correlation between RS to faces and CFMT scores (covarying out CCMT scores) across all participants in (*A*) medial prefrontal cortex, (*B*) left inferior parietal cortex, and (*C*) left dorsolateral prefrontal cortex. Activation maps are overlaid on a standard anatomical template image (ch2better.nii) using in MRIcron (http://www.mccauslandcenter.sc.edu/mricro/mricron/). All maps are thresholded at *t* = 3.46, *P *< 0.001 (10 contiguous voxels).

Next, to explore the relationship between RS and face memory we performed a whole-brain regression analysis (covarying out car memory performance). This revealed a positive relationship between CFMT scores and RS in several regions (Table 3; *P* < 0.001 uncorrected), with 3 large clusters apparent in left/medial prefrontal cortex (Fig. 4*A*), left inferior parietal cortex (Fig. 4*B*), and left dorsolateral prefrontal cortex (Fig. 4*C*) (see Supplementary Fig. 3 for regression plots. Note that these plots are presented for information only, and that these data were not subject to secondary statistical analysis; Vul et al. 2009). Activation in these regions remained significant at the same threshold after covarying out accuracy on the post-scan identity task. There was no evidence of a negative relationship between CFMT scores and RS even at a liberal threshold (*P *< 0.01 uncorrected).

**Table 3.** MNI coordinates of regions showing a significant positive correlation between RS to faces and CFMT scores across all participants (partialling out CCMT scores). All voxels significant at *P *< 0.001 uncorrected (10 contiguous voxels) at a whole brain level

Brain region	Hemisphere	*X*	*Y*	*Z*	Cluster size	*T*
Inferior parietal cortex	L	−30	−64	34	102	5.26
Medial prefrontal cortex	L	−24	26	−14	132	5.07
Dorsolateral prefrontal cortex	L	−46	42	18	103	5.02
	L	−54	34	6	14	3.85
Ventromedial prefrontal cortex	R	2	26	−22	20	4.12
Medial temporal cortex	R	42	−20	−22	14	4.09
Dorsomedial prefrontal cortex	L	−10	62	26	61	4.08
Anterior hippocampus	R	24	−6	−22	16	3.93

#### Eyetracking

Due to difficulties in tracking some participants’ pupils (e.g., drooping eyelids, corrective lenses), reliable eyetracking data were only available from 19 out of 30 participants (11 ASC). An independent-samples *t*-test revealed that mean dwell time on the upper region of the face did not differ between control and ASC participants (*P* = 0.81); Mean Dwell Time (SE): Controls = 861 ms (94.1); ASC = 771 ms (84.5). Closer inspection of the data revealed that one ASC participant appeared to show an abnormal pattern of gaze behavior, spending less than 3% of dwell time on the upper part of the face. After removing this participant from analysis, mean dwell time for the ASC group was 846 ms (43.0). We also re-analysed the ROI data after removing the same participant and found that the Repetition × Group interaction remained significant in right FFA (*P* < 0.05) and borderline significant in left FFA (*P* = 0.07). In addition, the correlation between RS and CFMT remained significant in all regions reported in Table 3 (*P* < 0.001 uncorrected).

An alternative explanation for group differences in RS is that groups differed in the extent to which they varied their gaze location within blocks (i.e., ASC participants may have looked around the faces more than controls). To address this, we calculated the standard deviation of fixation locations across trials within same- and different-identity blocks for each participant. Data were entered into an 2 × 2 ANOVA with Block (same-identity, different-identity) and Fixation variation (*x*,*y*), as within participants factors and Group as a between participant factor. This revealed no main effect of Group (*P* = 0.80) and no interactions between Block and Group (*P* = 0.39) or Block, Fixation, and Group (*P* = 0.11). Thus, groups did not differ in the extent to which they varied their gaze location within blocks.

### Experiment 2: RS to Geometric Shapes

#### ROI Analysis

**Figure 5.**
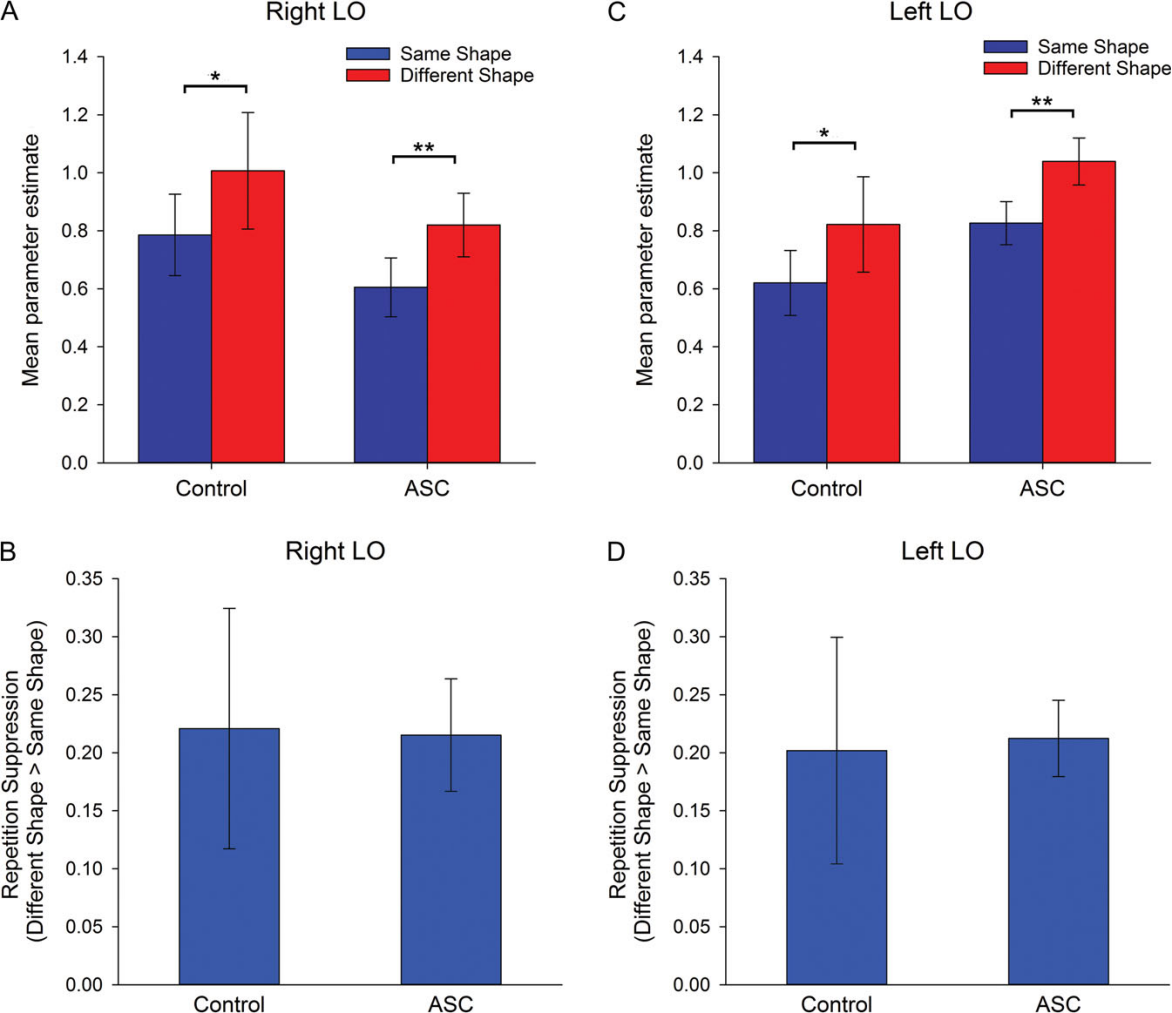
Experiment 2: RS to shapes. Mean parameter estimates (±1 SE) for same- and different-shape conditions (across image-size) in (*A*) right LO and (*C*) left LO, in control and ASC participants. RS (±1 SE) (i.e., different shape–same shape) in (*B*) right LO and (*D*) left FLO, in control and ASC participants. *P = 0.06, **P < 0.001.

ANCOVAs examined the effects of Repetition (same-shape, different-shape) and Image-Color (same-color, vary-color) as repeated measures, with Group (Control, ASC) as a between participants’ factor and scanner as a covariate. There was a significant effect of Repetition in right LO (*F*(1,24) = 15.8, *P* < 0.005, *η*_*ρ*_² = 0.40), reflecting a greater response in the different-shape condition relative to the same-shape condition (Fig. 5*A*), with no interactions between Repetition and Image-Color (*P* = 0.26) and no main effect of Group (*P *= 0.37) or Image-Color (*P* = 0.54). Crucially, there was no interaction between Repetition and Group (*P* = 0.96) or Repetition, Image-Color and Group (*P *= 0.11) in this region, indicating that the magnitude of RS did not differ between groups (Fig. 5*B*).

An ANCOVA revealed a significant effect of Repetition in left LO (*F*(1,25) = 18.34, *P* < 0.001, *η*_*ρ*_² = 0.42), with no interaction between Repetition and Image-Color (*P* = 0.49) (Fig. 5*C*). Again, we found no evidence of an interaction between Repetition and Group (*P* = 0.88) (Fig. 5*D*) or between Repetition, Image-Color, and Group (*P* = 0.13), and no main effect of Group (*P* = 0.20) or Image-Color (*P* = 0.65).

ANCOVAs for right and left pFs revealed a main effect of Repetition in both regions: rpFs: (*F*(1,19) = 8.92, *P *< 0.01, *η*_*ρ*_² = 0.32); lpFs: (*F*(1,24) = 11.01, *P* < 0.005, *η*_*ρ*_² = 0.31), reflecting a greater response in the different-shape condition compared with the same-shape condition (see Supplementary Fig. 4). The effect of Repetition was not modulated by an interaction with Image-Color (*P*’s > 0.77). We found no significant interaction between Repetition and Group, or between Repetition, Image-Color, and Group in either right pFs (*P*’s > 0.20) or left pFs (*P*’s > 0.46) (see Supplementary Fig. 4). There was no main effect of Group (*P*’s > 0.17) or Image-Color (*P*’s > 0.69.) in either region.

Analogous ANCOVAs including AQ as a covariate revealed no significant interaction between Repetition and AQ in any object-selective ROIs (*P*’s > 0.69). However, when considering control participants only, there was a borderline negative relationship between AQ and RS to shapes in rLO (*P* = 0.08).

#### Face-Selective ROIs

Finally, we examined RS to shapes within face-selective ROIs (see Supplementary Fig. 5). An ANCOVA revealed a significant effect of Repetition in both right FFA (*F*(1,22) = 8.88, *P *< 0.01, *η*_*ρ*_² = 0.29), and left FFA (*F*(1,17) = 14.17, *P *< 0.005, *η*_*ρ*_² = 0.46), and a significant effect of Group: right FFA: (*F*(1,22) = 9.89, *P* < 0.01, *η*_*ρ*_² = 0.31); left FFA: (*F*(1,17) = 5.65, *P* < 0.05, *η*_*ρ*_² = 0.25), with the ASC group showing a greater overall response to shapes than the control group. However, there was no Repetition × Group interaction in either region (*P*’s > 0.63). ANCOVAs for right and left OFA also revealed a main effect of Repetition in both regions: right OFA: (*F*(1,19) = 9.65, *P *< 0.01, *η*_*ρ*_² = 0.34); left OFA: (*F*(1,16) = 7.27, *P* < 0.05, *η*_*ρ*_² = 0.31), no effect of Group (*P*’s > 0.18), and no significant interaction between Repetition and Group (*P*’s > 0.65). Finally, there was no main effect of Repetition (*P *= 0.51) or Group (*P *= 0.18), and no interaction between Repetition and Group (*P *= 0.85) in right STS. Thus, reduced RS in FFA in the ASC group appears to be specific to faces rather than the consequence of “general” diminished RS in this region.

#### Whole-Brain Analysis

An independent samples *t*-test revealed no evidence of a group difference in RS to shapes (Different-Shape > Same-Shape) even at a liberal threshold (*P *< 0.01 uncorrected).

## Discussion

The aim of this study was to investigate RS to faces in adults with ASC, and to determine the neural mechanisms that underlie difficulties in face memory found in ASC. The results revealed that RS to faces in bilateral FFA was significantly reduced in ASC participants relative to age- and IQ-matched neurotypical controls. By contrast, there was no evidence of group differences in RS to geometric shapes in object-selective regions. ASC participants also performed significantly worse than controls on a standardized test of face memory but not on a test of car memory. Across participants, we found that face memory performance was positively correlated with RS in regions of left parietal and prefrontal cortex. These findings provide the first evidence that RS to facial identity is reduced in ASC. Moreover, they suggest that face memory abilities are linked to RS in regions commonly recruited during short-term and working memory tasks (Owen et al. 2005; Xu and Chun 2006), suggesting face memory difficulties in ASC may be a consequence of differences in the storage and/or maintenance of face representations across these regions.

Previous work investigating face processing in ASC has focused on the role of the FFA, with mixed evidence regarding extent to which the magnitude of this region's response differs between groups (Schultz et al. 2000; Pierce et al. 2001; Hall et al. 2003; Hadjikhani et al. 2004; Pierce et al. 2004; Dalton et al. 2005). In the current study, we found no evidence of a group difference in the overall magnitude of the FFA response, instead we found a group difference in the extent to which repetition of a face was associated with attenuation of the FFA response. Given that RS to shapes in FFA did not differ between groups, it seems unlikely that this finding reflects a difference in the general adaptive properties of this region. Moreover, given that the RS paradigm used here involved brief presentations of faces separated by a delay, similar to behavioral tests of face recognition memory, our results appear to accord with evidence of face-identity recognition difficulties in ASC in tasks that include a delay between target and test stimulus (Weigelt et al. 2012).

Previous work has found that individuals with ASC show reduced habituation to faces in bilateral amygdala but no group difference in habituation in the fusiform gyrus (Kleinhans et al. 2009). However, it is important to note that Kleinhans et al. did not measure RS as it is typically defined, as they did not compare the response to repeated presentations of the same face relative to presentation of different faces. Instead they measured the change in response between the first and second run of the same scanning session. In this sense, their approach is comparable to studies measuring amygdala habituation to facial expressions (Breiter et al. 1996; Phillips et al. 2001), rather than RS studies.

One possible explanation for the finding of reduced RS to faces in ASC participants is that it may reflect a difficulty in detecting changes in facial identity across trials. For example, previous work indicates that ASC participants show “reduced” neural sensitivity in FFA to small changes in face shape relative to that shown by neurotypical participants in an earlier study (Jiang et al. 2006) (although it should be noted they did not directly compare the 2 groups), while age-related reductions in RS have been shown to be related to the ability to distinguish between morphed versions of the same face (Goh et al. 2010). However, unlike the aforementioned studies, we only used identical face images in same-identity blocks and distinct identities in different-identity blocks. In addition, post-scan behavioral data indicated that ASC participants were equivalent to controls in detecting changes in identity across trials. Importantly, a reduced ability to distinguish faces would be expected to lead to a lower response in the different-identity condition only (i.e., Goh et al. 2010), however such an effect was not apparent here.

ASC has been associated with increased time spent attending to the lower regions of a face, rather than the eyes (Klin et al. 2002; Pelphrey et al. 2002). Thus, one possibility is that reduced RS in ASC is a consequence of differences in attentional focus. However, we believe this is unlikely to explain our findings for a number of reasons. First, eyetracking data revealed no difference in the amount of time ASC and control participants spent looking at the upper region of the face. Second, there was no group difference in overall FFA activity to faces (see Dalton et al. 2005). Third, there were no group differences in response time or accuracy on the dot-detection task during the faces scan (see Supplemental Materials), suggesting both groups were attending to the faces as instructed. On a similar note, it is possible that ASC participants might attend to different features or components of a stimulus on successive presentations (Happé and Frith 2006). However, we found no group difference in variation in gaze locations within blocks, and in Experiment 2, the results revealed that varying the color of the shape across trials did not lead to reduced RS in the ASC group.

An additional aim of this study was to determine whether reduced RS is a general characteristic of individuals with ASC. Recent proposals have characterized atypicalities in autism as a “disorder of prediction” (Sinha et al. 2014) or “attenuated use of prior knowledge” (Pellicano and Burr 2012), while predictive coding theories propose that RS is a consequence of a decrease in prediction error, that is, difference between bottom-up (stimulus-based) and top-down (prediction-based) inputs (Henson 2003; Friston 2005), mediated via “top-down” suppression of “errors” (Summerfield et al. 2008; Ewbank et al. 2011, 2013). Previously, we found a negative relationship between autistic traits and RS to faces in FFA (Ewbank et al. 2014). The results of the current study accord with this finding, suggesting reduced RS to faces is also apparent in individuals with a clinical diagnosis of an ASC. As in the previous study, we again found the relationship between RS and AQ was restricted to FFA and did not extend to other face-selective regions. Interestingly, while we found no group difference in RS in face-selective STS, ASC participants showed a greater overall response to faces in this region. What underlies this effect is unclear; however, previous work has shown that this region shows RS to gaze direction (Calder et al. 2007). Thus, one possibility is that a decreased response in controls reflects greater RS to a repetition of a constant gaze direction across blocks. Given the absence of a comparable averted gaze condition, however, this explanation remains purely speculative.

In the current study, we found no evidence of reduced RS to shapes in the ASC group. This contrasts with our previous finding of reduced RS as a function of increased autistic traits in a neurotypical population (although here a similar relationship was apparent when considering neurotypicals only). The reason for this discrepancy is unclear, and more research is needed to clarify whether the previous relationship replicates in independent studies. More importantly, this finding provides a challenge for the notion of a general deficit in predictive mechanisms in ASC, and accords with recent work challenging the notion of “attenuated priors” in ASC (Pell et al. 2016). Previous evidence also indicates that RS in visual cortex does not differ between ASC participants and controls when viewing repeated hand movements (Dinstein et al. 2010). While predictive-coding theories emphasize that “top-down” predictions are a general mechanism underlying RS, it is possible that “top-down” influences may play a differential role in RS to complex stimuli, such as faces, compared with relative simple stimuli such as geometric shapes. Indeed, evidence indicates that “predictive” modulation of RS in occipitotemporal cortex appears dependent upon the type and familiarity of the stimulus (Kovacs et al. 2013; Grotheer and Kovacs 2014). On a similar note, it has been proposed that individuals with ASC may find prediction harder in “non-systemisable” domains (e.g., social), than in highly lawful, systematic ones (Baron-Cohen 2006). While the design used here was identical to that used in previous work (Ewbank et al. 2014), a limitation of this paradigm is that we are unable to directly compare RS to faces and RS to shapes given that changes in the face and shape conditions were not fully equated; the size and color of shapes was varied, whereas only size of faces was varied. One possibility is that changes in color could be seen as a change in the “identity” of a shape, although it should be noted that we found no effect of color on RS to shapes, and previous work has indicated that RS is sensitive to change in the shape but not the color of objects (Cavina-Pratesi et al. 2010). However, future work will be needed to directly compare RS to faces and non-faces across equivalent image transformations.

An alternative explanation for the “face-specific” RS effect found here is that it reflects underlying differences in the representation of feature space in ASC. Qian and Lipkin (2011) have proposed that ASC is characterized by an atypical learning style, in which stimulus features are coded by separate, narrow, and non-overlapping tuning functions. They propose that ASC and typical learning styles favor low- and high-dimensional feature spaces, respectively. Face recognition, involves representation across a high-dimensional feature space (e.g., the size and shape of features and spatial relationships among them), whereas the processing of geometric shapes involves a relatively low feature space. However, the extent to which narrow tuning is associated with ASC remains to be determined.

Weigelt et al. (2012) have proposed that difficulties in face-identity recognition in ASC are primarily found on tasks that involve a memory component, whereas measures of face identity “perception” appear relatively intact. While previous work using standardized face memory tasks has reported reduced performance in ASC groups (O'Hearn et al. 2010; Kirchner et al. 2011), this work did not included equivalent tests of non-face memory. Here, we that showed that individuals with ASC showed a selective reduction in performance on a test of face memory relative to an equivalent measure of car memory as well as intact performance on a face discrimination task. This finding accords with work showing that children with ASC show deficits in memory for faces but not cars, as well as intact performance on a test of face perception (Weigelt et al. 2013). While our face discrimination task results suggest intact face-identity discrimination in ASC, it should be acknowledged that the ceiling effects observed here reflect the relatively simple nature of this task (i.e., determining whether a face has changed or not), thus the extent to which this task measures face perception abilities is unclear. Future work will be needed to provide a more rigorous test of the proposed dissociation between perception and face memory abilities in ASC (Weigelt et al. 2012).

We also investigated the relationship between individual variation in face memory and RS. An exploratory whole brain analysis revealed that performance on a face memory task was positively related to RS in regions thought to form part of the brain's working memory network (Owen et al. 2005); including inferior parietal cortex and dorsolateral prefrontal cortex. These regions have been linked to short-term storage (see Smith and Jonides 1998) and maintenance/manipulation of information, respectively (Miller et al. 1996), with evidence indicating that parietal cortex is involved in holding behaviorally relevant visual representations of faces (Jeong and Xu 2016). Furthermore, the relationship between RS and face memory could not be accounted for by variation in car memory performance. Interestingly, the relationship between face memory and RS was only apparent in left hemisphere regions commonly associated with storage of verbal rather than visual materials (Smith and Jonides 1999). Although participants did not perform an explicit memory task during the scan, one possibility is that individuals with ASC are less likely to use implicit verbal codes when viewing faces. This would accord with previous work indicating that ASC participants show reduced activity in left ventrolateral and dorsolateral prefrontal cortex during a working memory face task (Koshino et al. 2008). It should be noted that the results of our whole brain analysis were obtained using an uncorrected threshold (*P *< 0.001) and that future research is needed to determine the reliability of these findings. Finally, although the car memory test provides a measure of non-face memory matched with the CFMT, it does not provide a good behavioral correlate of RS to shapes. Thus, we are unable to determine whether RS to shapes is also related to variation in shape memory in similar or different regions to that found for faces.

In conclusion, we found that adults with ASC showed reduced memory for faces but not cars, and reduced RS to faces but not shapes. Furthermore, individual variation in face memory was related to RS in regions of parietal and prefrontal cortex. These findings accord with evidence suggesting ASC is associated with difficulties in face memory, and suggest difficulties in face memory may reflect differences in the way faces are stored and/or maintained across a network of regions involved in both visual perception and short-term/working memory.

## Supplementary Material

Supplementary Data

Supplementary Data

Supplementary Data

Supplementary Data

Supplementary Data

Supplementary Data

Supplementary Data
