# Supplementary figures and images for: Repetition Suppression and Memory for Faces is Reduced in Adults with Autism Spectrum Conditions
Source: Cereb Cortex. 2016 Nov 30;27(1):92–103. doi: 10.1093/cercor/bhw373 (PMC6044360; doi:10.1093/cercor/bhw373)

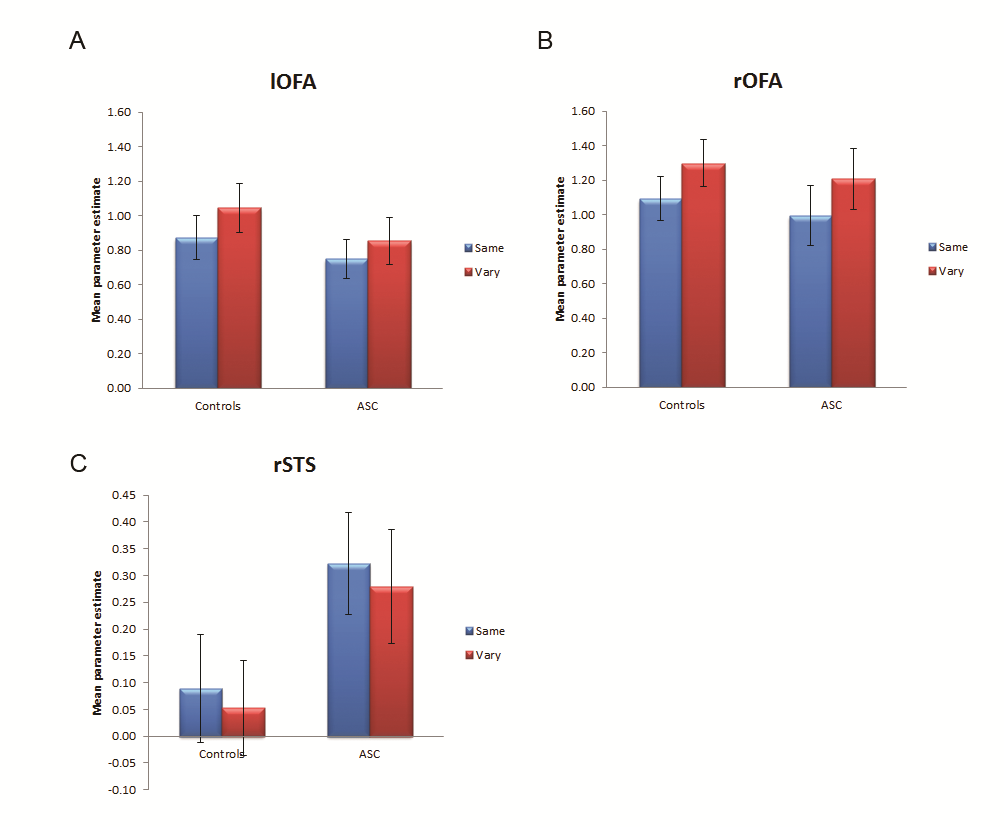

Supplement: Supplementary Data [file bhw373_figure_s1.png]

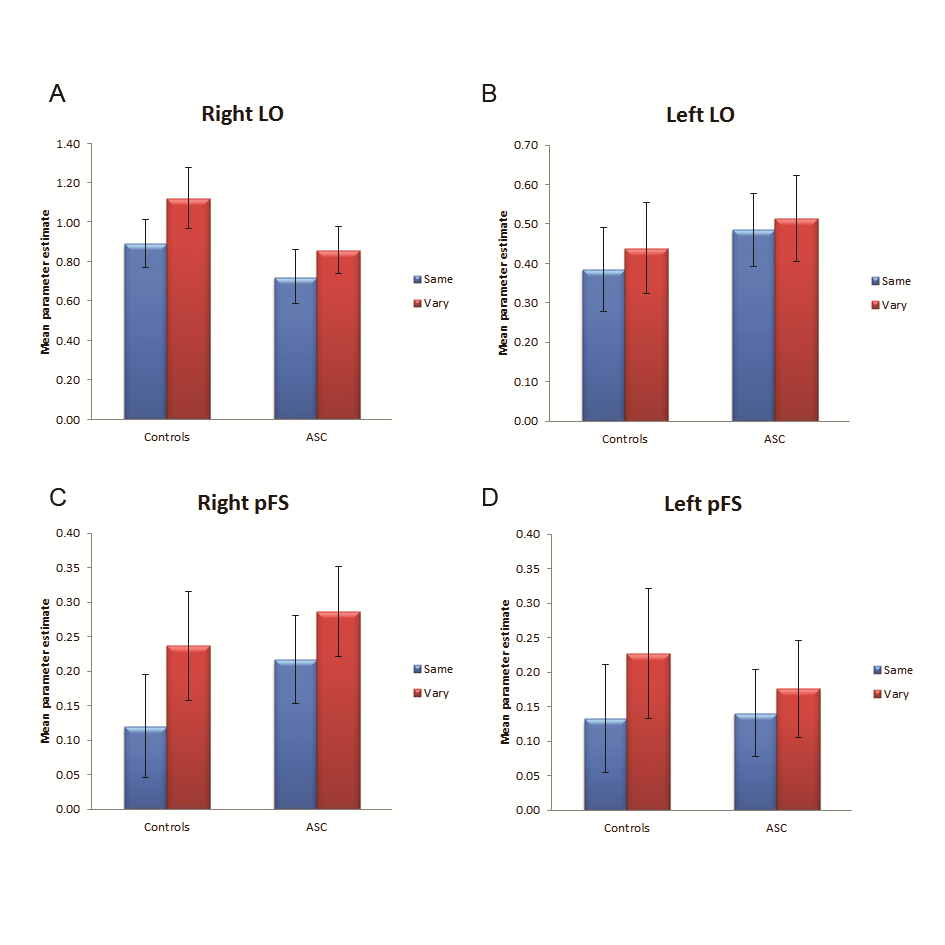

Supplement: Supplementary Data [file figure_s2.png]

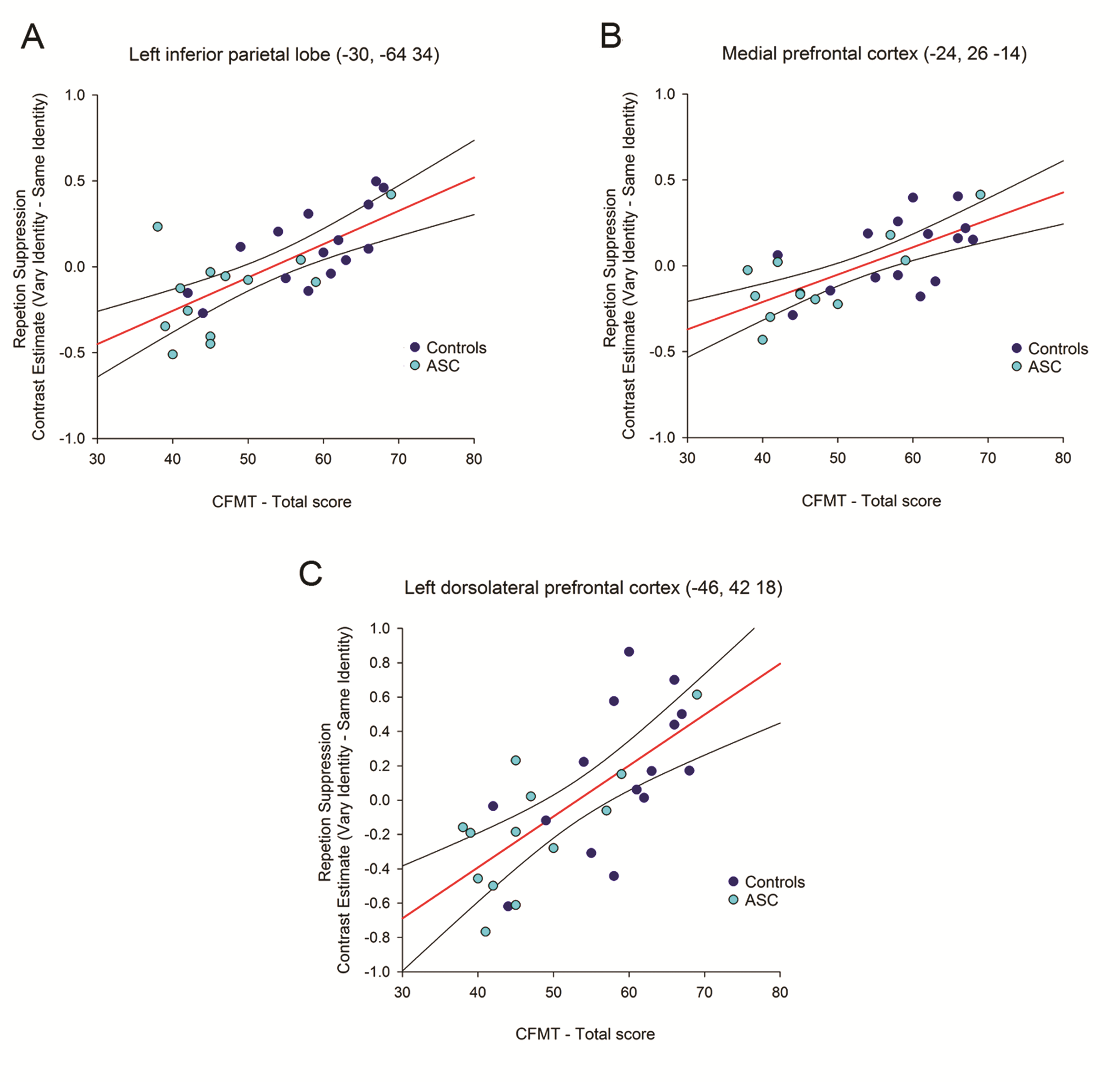

Supplement: Supplementary Data [file figure_s3.png]

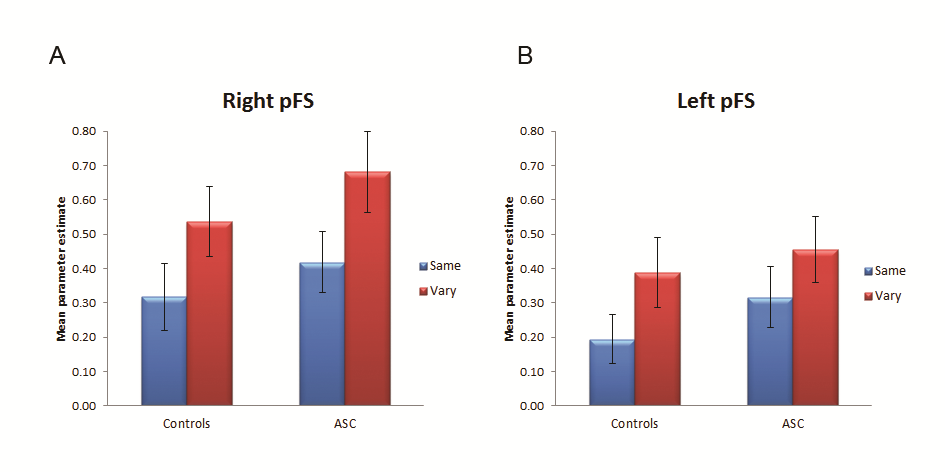

Supplement: Supplementary Data [file figure_s4.png]

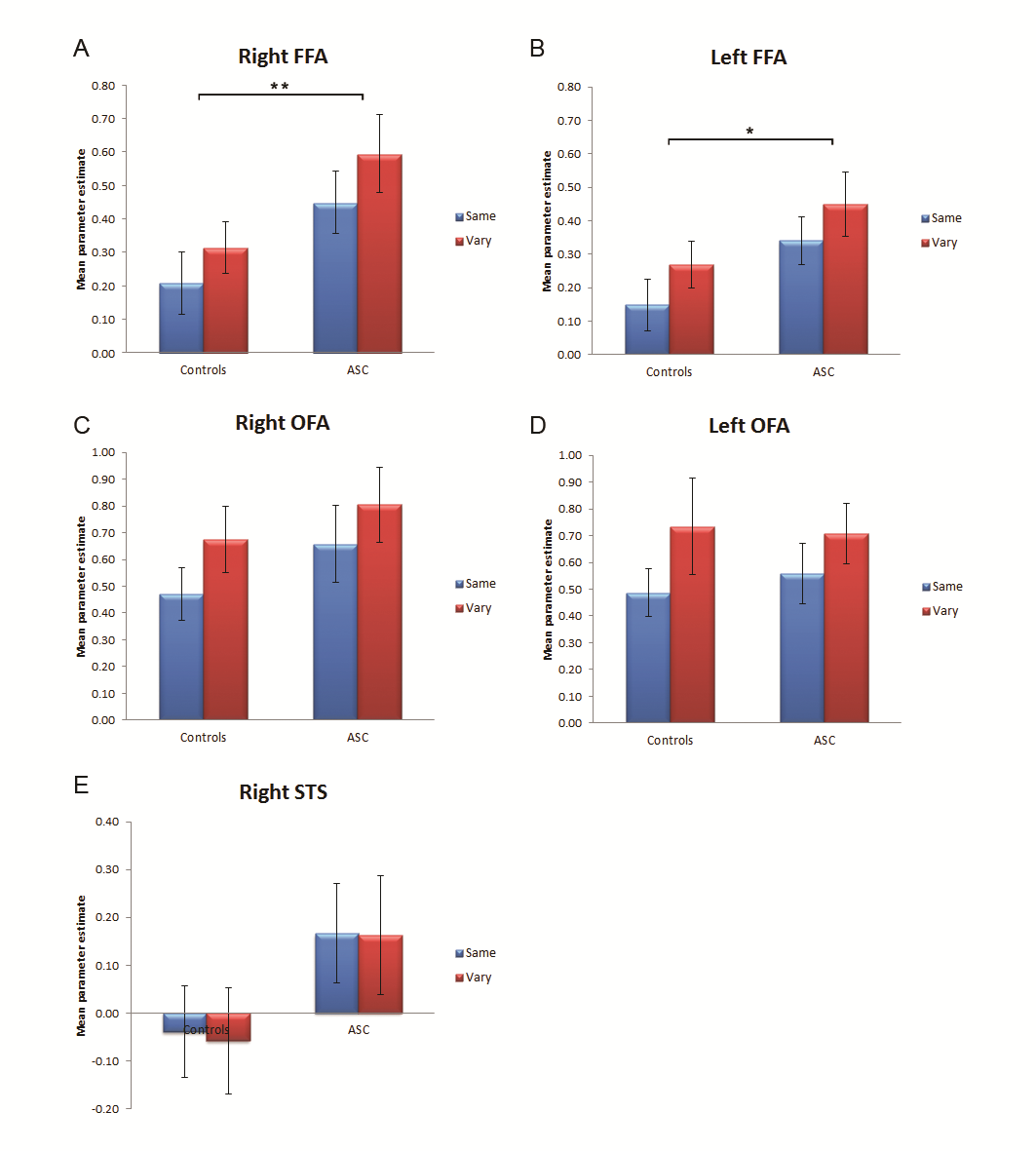

Supplement: Supplementary Data [file figure_s5.png]
