# Supplementary material for: Repetition Suppression and Memory for Faces is Reduced in Adults with Autism Spectrum Conditions
Source: Cereb Cortex. 2016 Nov 30;27(1):92–103. doi: 10.1093/cercor/bhw373 (PMC6044360; doi:10.1093/cercor/bhw373)
Supplement: Supplementary Data [file ewbank_et_al_supplemental_material_cc.doc]

**Results**

*Experiment 1: Dot detection task*

*A*ccuracy rates for the dot-detection task were high for both groups: Accuracy (SD): Control=97% (.05); ASC=98% (.06). Data were arcsine transform and entered into a 2x2x2 mixed measures ANOVA with Identity (Same, Different) and Image-Size (Same size, Vary Size) included as within participant measures and Group as the between participant measure. This revealed no significant effect of Group (p=.48) and no interactions between Identity and/or Image-Size and Group (p’s>31). The only significant effect was an interaction between Identity and Image-Size (F(1,29)=5.01, p=.033, *η_ρ_*²=.15), indicating that for both groups, accuracy decreased slightly when both identity and size varied (Accuracy (SD): Control = 94% (.09); ASC = 97% (.09).

Mean RT (SD) across all conditions was similar for the two groups RT (SD): Control = 479ms (42.6); ASC = 492ms (83.3). Data were subject to a 2x2x2 mixed measured ANOVA with Identity (Same, Different) and Image-Size (Same size, Vary Size) included as within participant measures and Group as the between participant measure. The only significant effect was an interaction between Image-Size and Group (F(1,29)=6.30, p=.018, *η_ρ_*²=.18), which indicates that whereas RTs were faster in the Same Size condition vs. the Vary Size condition for controls the (472ms vs. 485ms) there is a trend in the opposite direct for the ASC group (495ms vs. 488ms).

*Experiment 2: Dot detection task*

*A*ccuracy rates for the dot-detection task were high for both groups: Accuracy (SD): Control=98 % (.03); ASC=98 % (.03). Data were arcsine transform and entered into a 2x2x2 mixed measures ANOVA with Identity (Same, Different) and Colour (Same Colour, Vary Colour) included as within participant measures and Group as the between participant measure. There was no significant effect of Group (p=.66) and no interactions between Identity and/or Colour and Group (p’s>12). Mean RT (SD) across all conditions was similar for the two groups RT (SD): Control = 522ms (55.4); ASC = 513ms (58.01). Data were subject to a 2x2x2 mixed measured ANOVA with Identity (Same, Different) and Image-Size (Same size, Vary Size) included as within participant measures and Group as the between participant measure. There were no significant main effects or interactions (p’s>.39).
