# Supplementary material for: Repetition Suppression and Memory for Faces is Reduced in Adults with Autism Spectrum Conditions
Source: Cereb Cortex. 2016 Nov 30;27(1):92–103. doi: 10.1093/cercor/bhw373 (PMC6044360; doi:10.1093/cercor/bhw373)
Supplement: Supplementary Data [file ewbank_et_al_supplementary_legends.docx]

**Supplemental Figure 1.** Experiment 1: RS to faces in OFA an STS. Mean parameter estimates (+1SE) for Same- and Different-Identity conditions (across image-size) in (A) right OFA, (B) left OFA, and (C) right STS, in control and ASC participants. *p<.05.

**Supplemental Figure 2.** Experiment 1: RS to faces in object-selective ROIs. Mean parameter estimates (+1SE) for Same- and Different-Identity conditions (across image-size) in (A) right LO, (B) left LO, (C) right pFS, and (D) left pFS (excluding face-selective voxels), in control and ASC participants.

**Supplemental Figure 3.** Experiment 1: Relationship between face memory and repetition suppression across both ASC and control participants in (A) left inferior parietal cortex (B) medial prefrontal cortex and (C) left dorsolateral prefrontal cortex. All scatter plots show standardized residuals of contrast estimates of repetition suppression (covarying out performance on Cambridge Car Memory Test) plotted against Cambridge Face Memory Test (CFMT) scores. Regression line and 95% confidence intervals are shown.

**Supplemental Figure 4.** Experiment 2: RS to shapes in face-selective ROIs. Mean parameter estimates (+1SE) for Same- and Different-Shape conditions (across image-size) in (A) right FFA, (B) left FFA, (C) right OFA, (D) left OFA, and (E) right STS, in control and ASC participants. *p<.05, **p<.001.

**Supplemental Figure 5.** Experiment 2: RS to shapes in pFS. Mean parameter estimates (+1SE) for Same- and Different-Shape conditions (across image-size) in (A) right pFS and (B) left pFS, in control and ASC participants.
